# Ovine *HSP90AA1* Expression Rate Is Affected by Several SNPs at the Promoter under Both Basal and Heat Stress Conditions

**DOI:** 10.1371/journal.pone.0066641

**Published:** 2013-06-24

**Authors:** Judit Salces-Ortiz, Carmen González, Natalia Moreno-Sánchez, Jorge H. Calvo, M. Dolores Pérez-Guzmán, Magdalena M. Serrano

**Affiliations:** 1 Dpto. Mejora Genética animal. Inst. Nac. Invest. Agrarias y Alimentarias, Madrid, Spain; 2 ARAID, Zaragoza, Spain; 3 CERSYRA, Valdepeñas, Spain; University of South Florida College of Medicine, United States of America

## Abstract

The aim of this work was to investigate the association between polymorphisms located at the *HSP90AA1* ovine gene promoter and gene expression rate under different environmental conditions, using a mixed model approach. Blood samples from 120 unrelated rams of the Manchega sheep breed were collected at three time points differing in environmental conditions. Rams were selected on the basis of their genotype for the transversion G/C located 660 base pairs upstream the gene transcription initiation site. Animals were also genotyped for another set of 6 SNPs located at the gene promoter. Two SNPs, G/C_−660_ and A/G_−444_, were associated with gene overexpression resulting from heat stress. The composed genotype CC_−660_-AG_−444_ was the genotype having the highest expression rates with fold changes ranging from 2.2 to 3.0. The genotype AG_−522_ showed the highest expression levels under control conditions with a fold change of 1.4. Under these conditions, the composed genotype CC_−601_-TT_−524_-AG_−522_-TT_−468_ is expected to be correlated with higher basal expression of the gene according to genotype frequencies and linkage disequilibrium values. Some putative transcription factors were predicted for binding sites where the SNPs considered are located. Since the expression rate of the gene under alternative environmental conditions seems to depend on the composed genotype of several SNPs located at its promoter, a cooperative regulation of the transcription of the *HSP90AA1* gene could be hypothesized. Nevertheless epigenetic regulation mechanisms cannot be discarded.

## Introduction

Current concern about global warming and its effects over agricultural and livestock production systems have opened novel scientific opportunities to study the adaptation of organisms to new and harsher environmental conditions. In this context, the study of the genetic basis of traits linked with adaptation and fitness has great importance. Heat is one of the main sources of stress which has an important impact in livestock production. The genetic variability underlying animal’s thermo tolerance could be exploited in livestock breeding programs to achieve animals that could cope with the effects of heat stress over productive and functional traits. Among the livestock animals, sheep (*Ovis aries*), is one of the oldest domesticated species [1]. It is widely distributed throughout the world due to its high plasticity and adaptability to withstand poor nutrient diets and tolerance to extreme climatic conditions [2], [3]. Sheep is thus an interesting biological material to study the genetic basis of thermo-tolerance. There is some literature about heat stress effects over physiological and productive traits in cattle [4], [5], [6] and sheep [7], [8], [9], [10]. Also, at the molecular level, genes involved in the heat stress response have been described [11], [12], [13], [14]. Among them, those encoding heat shock proteins have been the most studied. However, in sheep there are few works regarding this topic.

Heat shock proteins (HSPs) [15], play a fundamental role in the maintenance of cellular homeostasis, under both physiological and stress conditions [16]. HSPs are organized into several families according to their molecular size (kDa). HSPs are highly conserved across species [17], particularly the 90 kDa heat shock protein (HSP90) [18]. In eukaryotes there are two major isoforms of HSP90 constituted by gene duplication [19]: the inducible form, HSP90α and the constitutive form, HSP90β. The *HSP90AA1* ovine gene (DQ983231) which encodes the *HSP90α* protein has been sequenced, mapped and characterized in sheep by Marcos-Carcavilla et al. [20]. In their work 34 polymorphisms (12 in the coding region, 14 in the promoter -Figure S1- and 8 in the intron 10) were detected. Further on, also a new INDEL (insertion/deletion) was observed at the promoter [21] (Figure S1). The transversion G/C located at position −660 in the gene promoter was associated with resistance/susceptibility to *scrapie* [22], with sperm DNA fragmentation in rams [23] and with the adaptation pattern of different sheep breeds to the thermal conditions in where they are reared [24]. In this last study, this polymorphism was associated with differences in the transcription rate of the *HSP90AA1* gene, being either the causal mutation which putatively modifies a transcription factor binding site or in linkage disequilibrium with the causal mutation. However, the study was based on a limited number of animals and on standard basic statistical methods used to analyze quantitative real-time PCR (qPCR) data and to asses differences in expression levels of alternative genotypes under control and heat stress conditions.

In general, qPCR has provided a powerful tool for quantifying gene expression. Nevertheless, it makes necessary to carefully consider some technical and analytical factors to ensure reproducible and accurate measurements and not lead to misinterpretations [25]. However, commonly, several of these essential procedures have been widely ignored. Those technical factors include the initial sample amount, RNA recovery and RNA purity and integrity [26], [27] among others. Some factors considered as analytical are the selection of the suitable housekeeping gene(s) (HK), the experimental design [28], the statistical method used, etc.

Traditional statistical analyses have been restricted to pair-wise comparisons of treatments in which Cq (quantification cycle) values of GOIs (genes of interest) were previously normalized using standard HKs. This kind of approach does not allow to include technical and biological effects having influence over gene expression data. The joint analysis of GOIs and HKs data can lead to a better partition of such sources of variation [29] and allows checking HK stability and subsequent normalization of GOIs. Mixed model methodology makes possible this kind of approach, giving the possibility of including systematic and random effects, and interactions among them. They constitute a powerful tool in qPCR analyses including more than two treatments and multiple experimental factors [30], [31].

The objectives of this study were to 1) confirm gene expression differences observed for alternative genotypes of the G/C_−660_ transversion of the *HSP90AA1* gene promoter using more animals and a wider range of climatic conditions that those in [12]; 2) study the effect over the expression levels of another six polymorphisms (A/C_−601_, G/A_−528_, G/T_−524_, A/G_−522_, G/T_−468_ and A/G_−444_) selected on the basis of their population genotype frequencies (Table S1) and relative position regarding SNP G/C_−660_; and 3) to simultaneously select the best HK and analyze expression data using a mixed model statistical approach that includes technical and biological sources of variation.

## Materials and Methods

### Ethics Statement

The current study was carried out under a Project License from the INIA Scientific Ethic Committee. Animal manipulations were performed according to the Spanish Policy for Animal Protection RD 53/2013, which meets the European Union Directive 86/609 about the protection of animals used in experimentation. We hereby confirm that the INIA Scientific Ethic Committee, which is the named IACUC for the INIA, specifically approved this study.

Animals belong to an artificial insemination centre, were raised in small groups in different barns and fed according to their necessities.

### Linkage Disequilibrium Analysis and Haplotype Determination

#### Animal material, nucleic acid isolation, DNA amplification and SNPs genotyping

Peripheral whole blood samples were collected from 103 animals of the Manchega Spanish sheep breed in order to analyse linkage disequilibrium among the 7 SNPs of interest located at the *HSP90AA1* promoter. Animals were grouped in 48 parent-offspring trios. Trios consist of 10 sires, 48 dams and 1 offspring per pair (all females). Three of the dams were also daughters from another trio. Genomic DNA was extracted from lymphocytes according to the salting out procedure [32]. The polymerase chain reaction was performed from 100 ng of genomic DNA using CERTAMP complex amplifications kit chemistry (Biotools, Madrid, Spain) with specific primers (Forward: 5′CGAGGCTCTGGCAGGCACTTGTTG3′ and Reverse: 5′ GCCGCCGTTCCCA GCCCTACCT 3′). A 499 bp fragment of the promoter containing SNP_−660_ and 6 more SNPs (−601, −528, −524, −522, −468, −444) was obtained. The resulting PCR fragment was purified with ExoSAP-IT (USB Corporation, OH, USA) and sequenced with specific primers (shown above).

#### Linkage disequilibrium estimation

PLINK software [33] was used to estimate linkage disequilibrium among all pairs of the 7 SNPs measured as r^2^, the squared correlation based on genotypic allele counts [34]. Hardy-Weinberg equilibrium exact test and observed and expected heterozygosities for each SNP were also calculated using PLINK.

### Detection of Putative Transcription Factor (TF) Binding Sites in Ovine *HSP90AA1* Promoter

Putative TF binding sites were predicted using TESS [35] (keeping default settings) and ALGGEN-PROMO [36], [37] (limiting to mammal transcription factors) softwares.

### Expression Analysis

#### Animal material

In order to confirm the association of the *HSP90AA1* polymorphism (G/C_−660_) previously associated with the adaptation to different thermal conditions in sheep previously described [24], 428 unrelated rams of Manchega Spanish sheep breed were genotyped (same protocol and primers as described in [24]). All animals belonged to an artificial insemination centre, and therefore they were reared under the same environmental and management conditions. A total of 120 out of 428 rams were selected based on their genotype: 40 CC_−660_, 40 GC_−660_ and 40 GG_−660_. Genomic DNA from these 120 animals was used to genotype the previosly defined 499 bp amplicon of the *HSP90AA1* promoter. Genotype frequencies are shown in Table 1.

**Table 1:** Genotype frequencies of the SNPs located at the *HSP90AA1* promoter in 120 rams of Manchega sheep breed.

SNPs								
−660	−601	−528	−524	−522	−468	−444	N	Freq.%
CC	AC	AA	GT	GG	GT	GG	2	1.63
CC	AC	AA	TT	GG	TT	GG	1	0.81
CC	AC	AG	GT	GG	GT	GG	1	0.81
CC	CC	AA	GT	GG	GT	GG	1	0.81
CC	CC	AA	TT	AG	TT	GG	3	2.44
CC	CC	AA	TT	GG	GT	GG	1	0.81
CC	CC	AA	TT	GG	TT	AA	1	0.81
CC	CC	AA	TT	GG	TT	AG	1	0.81
CC	CC	AA	TT	GG	TT	GG	26	21.14
CC	CC	GA	TT	AG	TT	GG	2	1.63
CC	CC	GA	TT	GG	TT	GG	2	1.63
CC	CC	GG	TT	GG	TT	GG	1	0.81
GC	AC	AA	GT	GG	GT	GG	1	0.81
GC	AC	GA	GT	GG	GT	GG	4	3.25
GC	CC	AA	TT	GG	TT	AG	1	0.81
GC	CC	AA	TT	GG	TT	GG	2	1.63
GC	CC	GA	GT	GG	GT	GG	1	0.81
GC	CC	GA	TT	AG	TT	GG	5	4.07
GC	CC	GA	TT	GG	TT	GG	21	17.08
GC	CC	GG	TT	GG	TT	GG	4	3.25
GG	CC	AA	TT	GG	TT	AG	1	0.81
GG	CC	AA	TT	GG	TT	GG	1	0.81
GG	CC	GA	TT	GG	TT	AG	2	1.63
GG	CC	GA	TT	GG	TT	GG	9	7.32
GG	CC	GG	TT	GG	TT	GG	29	23.58

Peripheral whole blood samples from the 120 rams were collected in 3 time points, corresponding to different climatic conditions in a dry region of central Spain (Ciudad Real). The 3 time points were in March, when environmental temperature conditions are mild, and in July and August when heat stress temperatures occur. Hereafter, we will refer to the March collection as the control. The temperature humidity index (THI) equation proposed by Marai et al. [8] was used as another indicator of thermal stress. This index combines both temperature and relative humidity. The enviromental parameters for the 3 points in time are shown in Table 2.

**Table 2:** Climate parameters at day of samples collection, and average values of the five days previous to samples collection.

Time points	Treatment name	AvT	MaT	MiT	Rh/100	Rhmax/100	THIavr	THImax	AvT-5daysBC	MaT-5daysBC	Rh-5daysBC/100	Rhmax-5daysBC/100	THIavr-5daysBC	THImax-5daysBC
23/03/2010	control	11.6	19.9	3.8	0.69	0.93	11.87	19.77	12.7	19.1	0.70	0.89	12.86	18.95
05/07/2010	July	26.8	35.0	16.8	0.39	0.64	24.47	32.69	25.2	33.5	0.49	0.83	23.49	32.49
03/08/2010	August	24.7	34.4	16.6	0.49	0.89	23.08	33.74	27.2	36.4	0.39	0.72	24.78	34.52

From: Manzanares (Ciudad Real) Meteorological Station, coordinates 654m-38° 59′47N-03° 22′23W (http://crea.uclm.es/siar).

AvT = average temperature (°C).

MaT = maximum temperature (°C).

MiT = minimum temperature (°C).

Rh/100 = relative humidity (%)/100.

Rhmax/100 = maximum relative humidity (%)/100.

THIavr = THI calculated with the average temperature and relative humidity.

THImax = THI calculated with the maximum temperature and the maximum relative humidity.

AvT-5daysBC = Average mean temperature of the five days previous to collection (°C).

MaT-5daysBC = Average maximum temperature of the five days previous to collection (°C).

Rh-5daysBC/100 = Average relative humidity of the five days previous to collection (%)/100.

Rhmax-5daysBC/100 = Average maximum relative humidity of the five days previous to collection (%)/100.

THIavr-5daysBC = THI calculated with the average temperature and relative humidity of the 5 days before collection.

THImax-5daysBC = THI calculated with the average maximum temperature and maximum relative humidity of the 5 days before collection.

Temperature humidity index (THI) calculated as THI = T°C – ((0.31–0.31RH) (T°C-14.4). T = temperature in °C; RH = relative humidity in %/100 [8].

#### Total RNA isolation and cDNA synthesis

Total RNA was isolated from 9 ml of whole blood using the LeukoLock kit (Ambion, Inc., TX, USA), following manufacturers instructions. RNA concentration was determined using a NanoDrop ND-1000 UV/Vis spectrophotometer (Nanodrop Technologies, Inc., DE, USA). Degradation of RNA samples was assessed with the Agilent 2100 bionalyzer (Agilent Technologies Hewlett-Packard-Str.8 76337 Waldbronn, Germany) in RNA Nano Chips, following manufacturers instructions. RIN (RNA Integrity Number) values were obtained. cDNA was synthesized using the ImProm-II™ Reverse Transcription System (Promega Corp., WI, USA).

#### Quantitative reverse transcription polymerase chain reaction (qRT-PCR)

qRT-PCR was performed on all samples collected. Three HKs were tested, *MDH1*, *SDHA* and *HSP90AB1*. *MDH1* and *SDHA* became the most stable HK pair for the heat stress response in sheep under similar conditions [38]. Also the *HSP90AB1* gene was included as HK candidate since its expression is ubiquitous, less inducible and more constitutive than that of the *HSP90AA1* gene [39], [40]. Primers were designed with NetPrimer software (Biosoft International, CA, USA), and are listed in Table 3 together with amplicon sizes and CG content. Primers were designed avoiding possible genomic DNA amplifications. *In silico* specificity of the amplicons was screened by BLAST searches.

**Table 3:** Primers and efficiencies of the qPCR reactions.

Gene	Forward primer (5′-3′) Reverse primer (5′-3′)	Ampliconsize (bp)	Efficiencies	Amplicon % bases and GC content
HSP90AA1	CCACTTGGCGGTCAAGCATT AAGGAGCTCGTCTTGGGACAA	80	1.951	A/22.50 G/25.00 C/31.25 T/21.25GC content 47.50
MDH1	GGTCAAATTGCATATTCACTACTA ACCATCCAGGACACCCATCAT	117	1.883	A/23.07 G/20.51 C/32.48 T/23.93GC content 43.58
SDHA	GGCATCCCCACCAACTACA TACACCACCTCAAAGCCCCG	134	2.000	A/35.55 G/29.62 C/17.03 T/17.77GC content 65.17
HSP90AB1	TACATCACTGGTAAGAGCAAAGA TACACCACCTCAAAGCCCCG	81	1.950	A/37.03 G/18.52 C/22.22 T/22.22GC content 55.55

qPCR amplification reactions were performed from 100 ng of cDNA using LightCycler® 480 SYBR Green I Master kit (Roche, Switzerland). Reactions were run in triplicate on a LightCycler® 480 (Roche, Switzerland) following manufacturer’s cycling parameters. Dissociation curves were performed for each gene to check primer specificity and to confirm the presence of a unique PCR product. The corresponding mRNA levels were measured and analyzed by their Cq.

To estimate PCR efficiencies, standard curves based on 6 serial dilutions (1/20 from a departure concentration of 50 ng/µl) of a cDNA stock (a cDNA mixture of more than 121 samples accounting for the 3 genotypes and the 3 time points) were performed. Efficiencies (E) were calculated from the slope of curves as in Rasmussen and coworkers’ [41]. Estimated E for each gene are shown in Table 3.

### Statistical Procedures

#### Statistical analysis of RIN values

A mixed model was fitted by using the MIXED procedure of the SAS statistical package [42] for determining factors affecting RIN values. RIN value of all samples were included as a dependent variable. Fixed effects included were genotype G/C_−660_ (G) - 3 levels: CC, GC and GG -; date of collection (D) - 3 levels: Control, July and August -; group of sample processing (GP) - 4 levels corresponding to the barn where a group of animals were located and sampled - and the interaction date of collection x group of sample processing (DxGP) were included as fixed effects. The barn needs to be included because it is related to the period of time between samples collection and processing. The animal (A) was included as random effect. Goodness of fit statistics AIC (Akaike’s Information Criterion) and BIC (Schwarz’s Bayesian Criterion) were used as criteria for model selection. A type III fixed effects test was used to determine significance of the effects included in the model. *P*<0.05 was established as threshold for statistical significance.

#### HK selection

HK selection among *HSP90AB1, MDH1* and *SDHA* genes followed the strategy from Serrano et al. [38]. As amplification efficiencies of some genes were <2 (<100%), Cq data were transformed using the equation proposed by Steibel et al. [29] to rescale Cq values.

The equation of the mixed model used was the following:
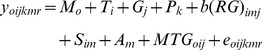
(1)where y_oijkmr_ is the transformed Cq data of the j^th^ gene, from the r^th^ well, in the k^th^ plate, collected from de m^th^ animal under the i^th^ treatment; M_o_ is the fixed effect of the o^th^ genotype; T_i_ is the fixed effect of i^th^ treatment; G_j_ is the fixed effect of the j^th^ gene; P_k_ is the effect of the k^th^ plate; b(RG)_imnj_ is the interaction between the RIN value of the mi^th^ sample and the j^th^ gene, where b is the regression coefficient of RIN x gene variable on Cq; S_im_ is the random effect of the biological sample 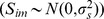; A_m_ is the random effect of the animal from where samples were collected 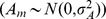; MTG_oij_ is the random interaction effect among the o^th^ genotype, the i^th^ treatment and the j^th^ gene 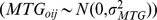; e_oijkmr_ is the random residual. Gene specific residual variance (heterogeneous residual) was fitted to the gene by treatment effect 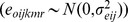.

Expression stability values were obtained by calculating the Mean Square Error (MSE), which was defined as in [38].

#### Analysis of expression results

Statistical analysis of gene expression was carried out following the method proposed by Steibel et al. [29]. As amplification efficiencies for *HSP90AA1*, *HSP90AB1* and *MDH1* genes were <2, Cq data were transformed as aforementioned. The mixed model fitted was:
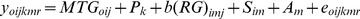
(2)where effects were as in model 1, except that in this case the MTG factor was included in the model as fixed effect and the residual variance was heterogeneous for the gene effect) .

To test differences, *diff_GOI_*, in the expression rate of alternative genotypes and to obtain fold change (FC) values from the estimated MTG differences, the approach suggested in [29] was used. Significance of *diff_GOI_* estimates was determined with the *t* statistic.

Also asymmetric 95% confidence intervals (up and low) were calculated for each FC value by using the standard error (SE) of *diff_GOI_*:
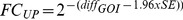
(3)

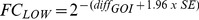
(4)


**Figure 1:**
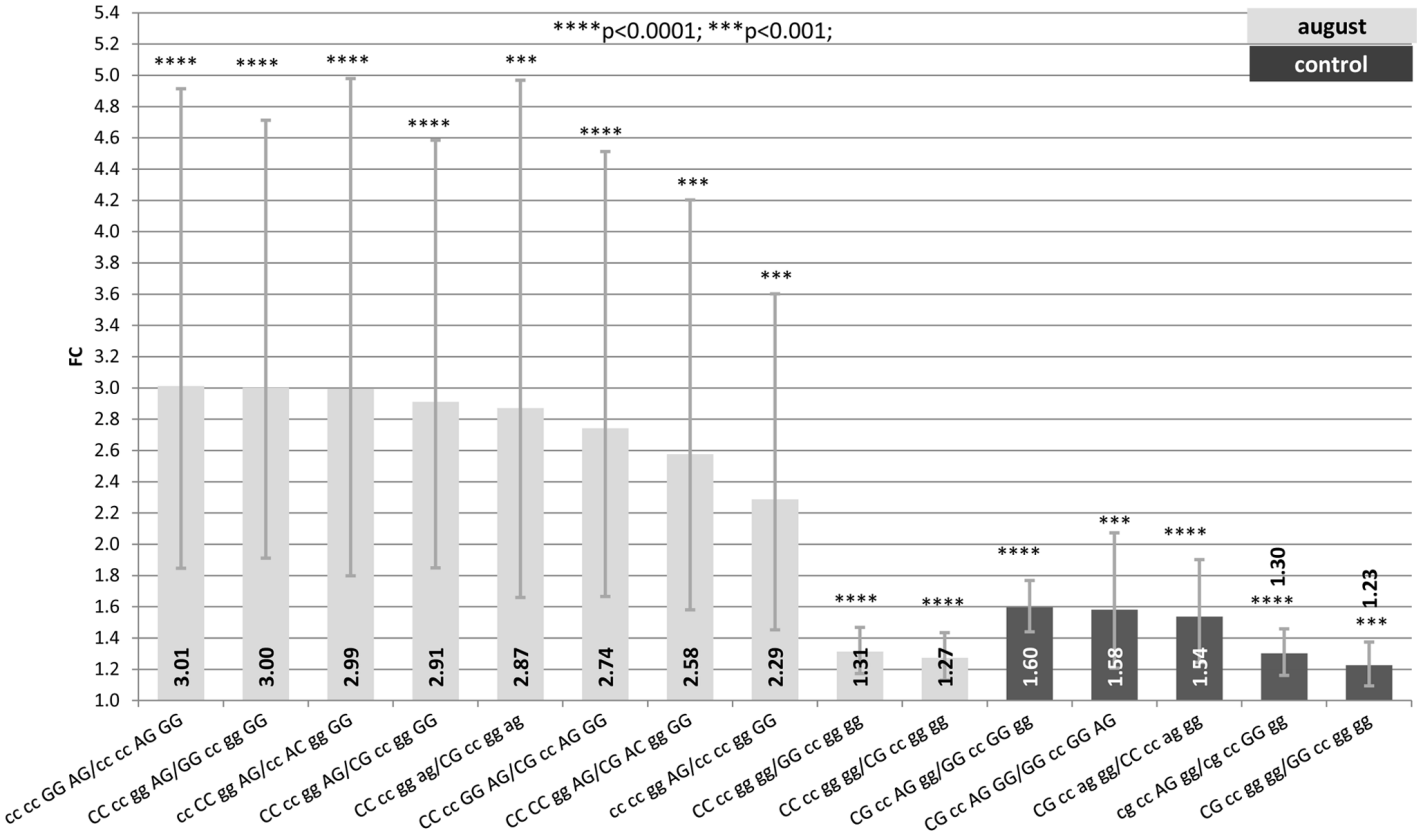
Fold change (FC) for the contrast among alternative genotypes G/C−**660**-C/A_−601_-G/A_−522_-A/G_−444_ of the *HSP90AA1* promoter within each treatment (Control, July and August) normalized by *HSP90AB1*. Segments indicate the 95% confidence interval (FC_up_-FC_low_). In abscissa the FC, in ordinate genotype contrasts. Asterisk over each bar indicates the significance level of the contrasts.

**Figure 2:**
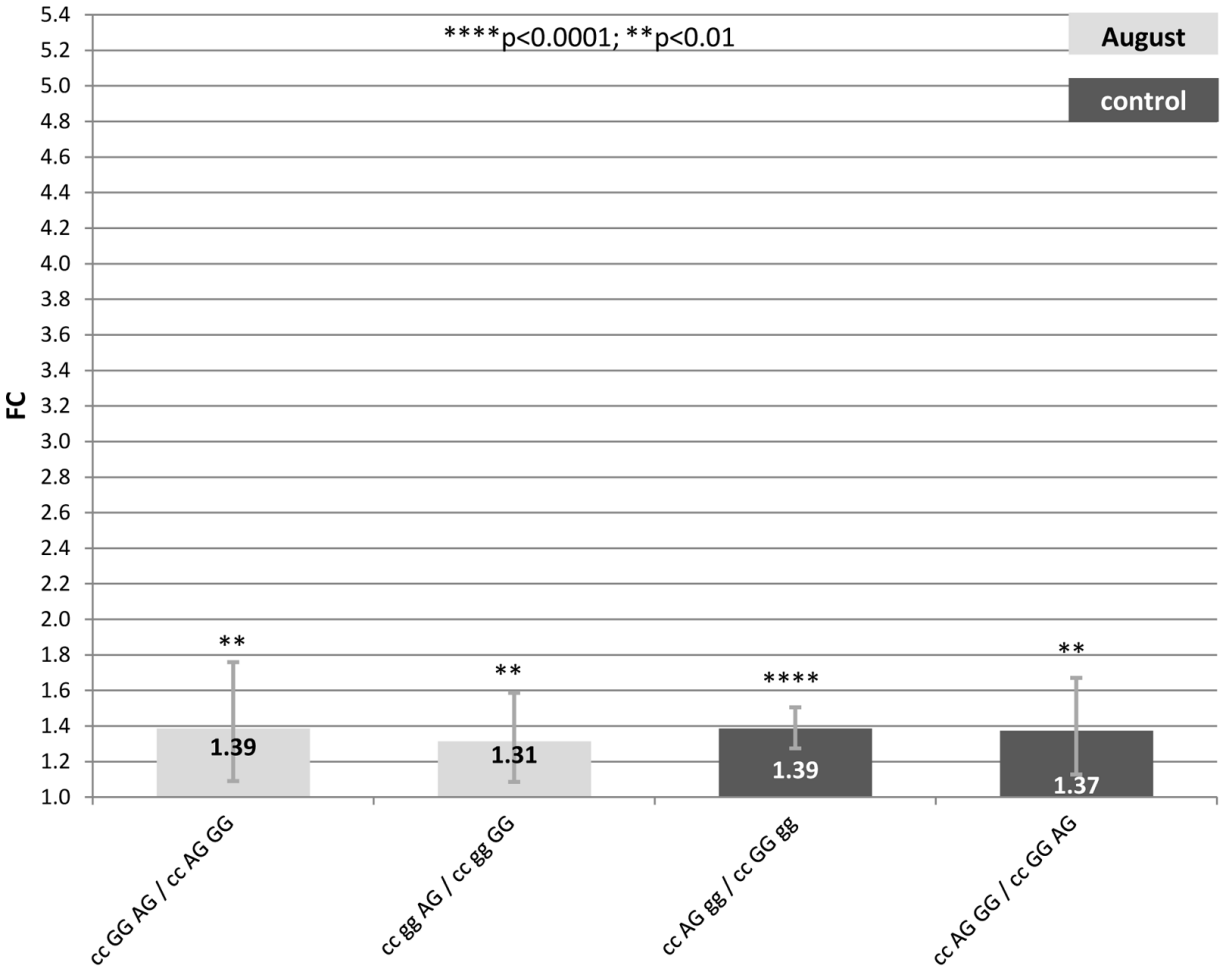
Fold change (FC) for the contrasts among alternative genotypes A/C_−601_-G/A_522_-A/G_−444_ of the *HSP90AA1* promoter within each treatment (Control, July and August) normalized by *HSP90AB1*. Segments indicate the 95% confidence interval (FC_up_-FC_low_).In abscissa the FC, in ordinate genotype contrasts. Asterisk over each bar indicates the significance level of the contrasts.

**Figure 3:**
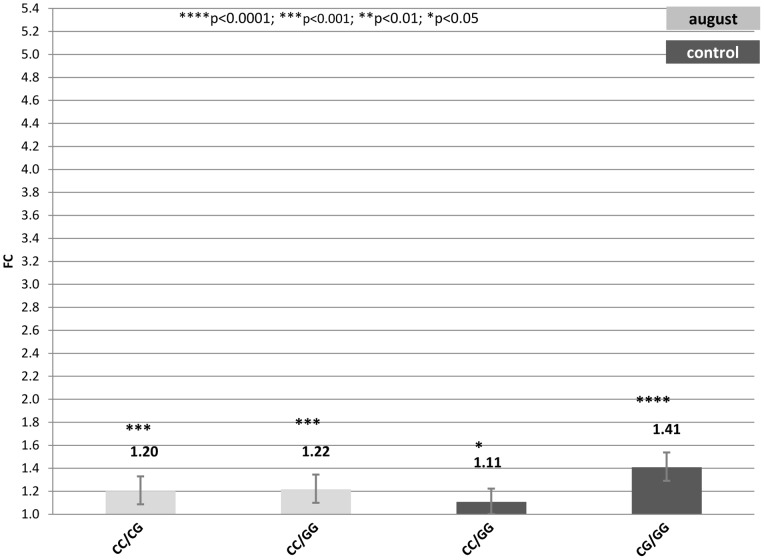
Fold change (FC) for the contrast among alternative genotypes G/C_−660_ of the *HSP90AA1* promoter within each treatment (Control, July and August) normalized by *HSP90AB1*. Segments indicate the 95% confidence interval (FC_up_-FC_low_).In abscissa the FC, in ordinate genotype contrast. Asterisk over each bar indicates the significance level of the contrasts.

Only contrasts between genotypes expression data that remained significant after the Holm-Bonferroni correction and with a FC >1 are going to be discussed. Supplementary Tables (Tables S2, S3 and S4) show estimates, standard errors, FC and confidence intervals (FC_up_-FC_low_) of significant contrasts between genotypes in each treatment. Also FCs are graphically represented in Figures 1, 2 and 3 where segments indicate 95% confidence interval.

## Results

### Linkage Disequilibrium Analysis

Results from Hardy-Weinberg equilibrium exact test and expected and observed heterozygosities are shown in Table S5. No deviations from the Hardy-Weinberg equilibrium were observed for any of the SNPs genotyped on population composed by trios. The average expected and observed heterozygosities were 0.256 and 0.306, respectively. The SNPs with the lowest allele frequencies were A/G_−522_ and A/G_−444_ (0.018 and 0.053, respectively).

Results from linkage disequilibrium analyses are shown in Table S6. The analysis revealed the existence of two blocks of SNPs segregating jointly. One block was constituted by the SNPs at positions −660 and −528 (r^2^ = 0.95), and the other was integrated by the SNPs mapped at −601, −524 and −468 (r^2^ between them ranged from 0.92 to 1.00). Linkage disequilibrium between other SNP pairs showed values lower than 0.06, ranging from 0.001 to 0.059.

### Statistical Analysis of RIN Values

The model including the interaction DxGP as a fixed effect and the animal as a random effect, showed the lowest values for the goodness of fit criteria (AIC and BIC). Estimated animal and residual variance were 0.11 and 2.25, respectively. Type III fixed effects test showed a highly significant (p<0.0001) effect of DxGP on RIN values but no significant effect were observed for G, D and GP on the trait.

Thus, as RIN values only depend on the order in which samples were processed after their collection, it can be included as a systematic effect in the statistical model used to analyse expression data.

### Best HKs

Given the linkage disequilibrium results for the 7 SNPs, the “genotype” included as an effect in the mixed model to test the stability of genes was G/C_−660_-A/C_−601_-A/G_−522_-A/G_−444_. Table 4 shows MSE values obtained for each gene within treatments and across genes. *HSP90AB1* was in all cases the most stable gene, followed by *HSP90AA1*. Therefore, *HSP90AB1* was selected as the only HK to normalize the expression results of *HSP90AA1*. The highest stability values for all genes corresponded to samples collected in August and lowest stability values corresponded to the control samples.

**Table 4:** Minimum square error (MSE) within and across treatments for *HSP90AA1, HSP90AB1, MDHA* and *SDHA* genes.

treatment	gene	MSE within treatment	MSE within gene
August	*HSP90AB1*	**2.51**	
August	*HSP90AA1*	3.17	
August	*SDHA*	3.84	
August	*MDH1*	9.81	
July	*HSP90AB1*	**3.27**	
July	*HSP90AA1*	6.07	
July	*SDHA*	7.46	
July	*MDH1*	9.92	
control	*HSP90AB1*	**4.62**	**4.62**
control	*HSP90AA1*	6.39	6.39
control	*MDH1*	8.20	9.92
control	*SDHA*	9.94	9.94

### Environmental Conditions and Statistical Analysis of Gene Expression

As it is shown in Table 2 the maximum temperatures for days of samples collection in July, 35.0°C, and August, 34.4°C, exceeded the sheep thermoneutral zone, but this is not the case for the average temperatures, 26.8°C and 24.7°C, respectively. For both time points, average and maximum THI values occurred in the zones of severe and extreme heat stress [43]. The THImax was one unit higher in August than in July. Also in August the THImax, calculated over the five days before collection, was two units higher than those of July. For the control time point, temperatures and THI values indicated no heat stress conditions.

**Figure 4:**
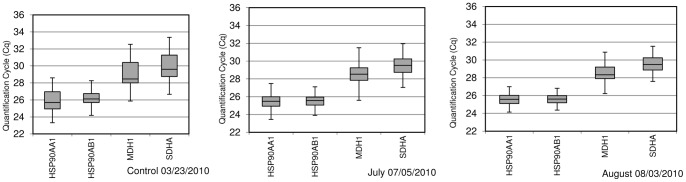
Distribution of Quantification Cycle (Cq) values for the target gene *HSP90AA1* and the reference genes *HSP90AB1*, *MDH1* and *SDHA*. They were obtained by qPCR from samples collected at three different time points (Control, July and August) along the year. Boxes show the range of Cq values within each gene and treatment; the centre line indicates the median; extended vertical bars show standard deviation of the mean.

Raw Cq values for all genes in each treatment are shown in Figure 4. Under control conditions, Cq values for the *HSP90AA1, HSP90AB1, MDH1* and *SDHA* genes were 26.2, 26.6, 29.4 and 30.3, respectively. Smaller Cq values were observed for all genes in samples collected under high temperatures (July and August). They were 25.7 for both chaperones and 28.8 and 29.7 for *MDH1* and *SDHA*, respectively. Variability in the expression rate of all genes was higher in samples collected under control conditions.

Based on linkage disequilibrium results, 3 sets of genotypes were selected to carry out expression studies. Genotypes considered in separate analyses were: 1) G/C_−660_-A/C_−601_-A/G_−522_-A/G_−444_, 2) A/C_−601_- A/G_−522_-A/G_−444_, 3) G/C_−660_.

#### Overall outcomes from fitting the mixed model

Test type III of fixed effects shows high significant F values (p<0.0001) for the MTG, P and b(RG) effects in all sets of genotypes. Estimates of the P effect levels (74) averaged 0.73 and ranged between 0.01 to 1.41 Cq. Regression coefficient estimates relating covariation of Cq and RIN values for each gene, were −0.28, −0.47, −0.55 and −0.67 for *HSP90AB1, HSP90AA1, MDH1* and *SDHA*, respectively.

Estimates of animal, sample and residual variances were very similar for the 3 sets of genotypes studied. Animal variance was very small and ranged between 0.03 and 0.05. Sample variance (1.40) was 2.8 times higher than the animal effect one. *HSP90AB1* showed a residual variance (0.0086) 34 times lower than the one of *HSP90AA1* (0.27) and 53 times lower than the one of *SDHA* (0.46). *MDH1* had the highest residual variance (1.34).

#### 1) Genotype G/C_−660_-A/C_−601_- A/G_−522_-A/G_−444_

Table S2 and Figure 1, show results for contrasts among G/C_−660_-A/C_−601_-A/G_−522_-A/G_−444_ genotypes under the different climatic conditions considered.

For samples collected in August, CC_−660_-CC_−601_-GG_−522_-AG_−444_ showed the highest expression rate (differences in FC from 2 to 3) when comparing with other 8 genotypes. Seven of these 8 genotypes were GG_−444_, highlighting the importance of this position for the expression rate of the gene under heat stress conditions independently of the other three SNPs. Two significant contrasts pointed out the effect of SNP_−660_ in terms of expression efficiency. CC_−660_ showed differences in FC of 1.27 and 1.31 when comparing with GC_−660_ and GG_−660_, respectively. It is important to note that contrasts showing differences in FC from 2.3 to 3.0 were those with wider confidence intervals which indicate higher estimated standard errors. This is due to the fact that in many of these contrasts, composed genotypes are at very low frequencies. In some cases only one animal exhibit the genotype compared. For comparisons where FC differences ranged between 1.2 and 1.3, confidence intervals were narrower due to the higher frequencies of the genotypes compared.

For samples collected in July, no comparisons between alternative genotypes were significant after the Holm-Bonferroni correction.

For the 5 significant contrasts of control samples the loose of the effect over the expression rate of the SNP_−444_ in the gene promoter and the change in the expression rank of the −660 genotypes were the most important effects observed. In 4 contrasts the GC_−660_-CC_−601_-AG_−522_-GG_−444_ genotype showed differences in FC from 1.3 to 1.6 when compared with alternative genotypes at −660 and −522. The superiority in terms of expression rate seems to depend in this case on the combination of genotypes existing at −660 and −522. Thus, the double heterozygous GC_−660_-AG_−522_ showed higher expression levels than GG_−660_-GG_−522_ (1.58–1.60), CC_−660_-AG_−522_ (1.54) and GC_−660_-GG_−522_ (1.30). Finally, the SNP_−601_ did not seem to be involved in the regulation of the basal expression of the gene. In this case, differences in the confidence intervals were smaller since genotypes compared had higher frequencies.

#### 2) Genotype A/C_−601_- A/G _−522_-A/G_−444_

To determine the impact of SNP_−660_ over the expression rate of the gene under the different environmental conditions previously described [24], we have tested a genotype of three SNPs excluding SNP_−660_.

Table S3 and Figure 2, show results for contrasts among A/C_−601_- A/G _−522_-A/G_−444_ genotypes under the different climatic conditions considered.

Regarding the high decrease in the magnitude of the FC differences for the 4 significant comparisons, we can confirm the critical influence of SNP_−660_ over the gene expression rate. Surprisingly, higher differences in FC were detected for contrasts in control than in August, revealing a more important effect of these SNPs on the basal expression of the *HSP90AA1* gene under control conditions.

In samples collected in August, the two significant contrasts with the highest expression rate implied the AG_−444_ genotype in all cases. Again, AG_−444_ showed higher expression rate (FC = 1.3–1.4) over GG_−444_ under heat stress conditions. Genotypes at −601 and −522 did not show any clear effect. No significant contrast was found among genotypes from samples collected in July.

Once more, under mild temperatures the SNP_−444_ lost its effect over the *HSP90AA1* expression which appeared under heat stress conditions. As in the case above mentioned, AG_−522_ showed a positive effect over the expression of the gene comparing with GG_−522_ (FC = 1.37–1.39).

#### 3) Genotype G/C_−660_

Table S4 and Figure 3, show results for contrasts among G/C_−660_ genotypes under the different climatic conditions considered.

Contrasts of this genotype showed lower differences in FC than that observed for the previous 2 sets of genotypes. Two comparisons were significant in samples collected in August. Differences in FC were 1.22 and 1.20 for the contrasts CC_−660_ vs. GG_−660_ and CC_−660_ vs. GC_−660_, respectively, showing the superiority of the CC genotype at position −660 over the other genotypes in terms of gene expression rate under heat stress conditions.

In July samples, no comparison had statistic significance. However, under control temperatures, 2 contrasts showed significant differences in FC, CC_−660_ vs. GG_−660_ with a FC equal to 1.11 and GC_−660_ vs. GG_−660_ with a FC of 1.41.

Thus, when considering only SNP_−660_, results indicated no differences in *HSP90AA1* expression rate across treatments, but the existence of such differences across genotypes.

In order to understand better the results obtained, contrasts involving SNP_−601_ and SNP_−522_, were carried out (Figure S2 and Figure S3). In the first analysis, alternative genotypes of A/C_−601_-A/G_−522_ were compared. Only one significant contrast was found under control conditions. A FC value of 1.4 was observed for the contrast CC_−601_-AG_−522_ vs. CC_−601_-GG_−522_, confirming the effect of A/G_−522_ over the basal expression of the gene under mild temperatures. SNP_−601_, did not seem to play any clear role in the *HSP90AA1* expression rate.

In the second additional analysis, genotypes of G/C_−660_-A/G_−522_ were compared to elucidate the relevance of SNP_−522_ under heat stress conditions. In this case, significant contrasts were found for control samples and those collected in August. The importance of SNP_−660_ in August was again revealed. CC_−660_ had higher expression rate than GC_−660_ and GG_−660_ (FC = 1.24 and 1.27, respectively). Under control conditions the effect of SNP_−522_ was clear (FC = 1.28 at least). Changes in the behavior of G/C_−660_ under control conditions were observed as well. In this case, GC_−660_ was superior to CC_−660_ and GG_−660_ as it was showed in previous analyses.

## Discussion

In this study, we aimed to confirm the results obtained previously [24], that showed an association of the SNP_−660_ of the ovine *HSP90AA1* gene promoter with the expression levels of this gene under different environmental temperatures, using a higher amount of samples. We also aimed to increase the information available of the biological process underlying this type of stress conditions. With these purposes we have studied new polymorphisms found at the promoter region, which would affect the expression rate of the gene not only in heat stress events as it was firstly thought, but also modulating its basal expression.

### RIN Effect

Best conservation and minimum degradation processes are critical points when sampling commercial livestock animals for expression studies. The degree of RNA degradation in the samples affects gene expression measurements. We have established that RIN values depended neither on the source of biological sample (the animal) nor on the environmental conditions surrounding samples collection. The only factor having a significant effect on RIN values was the period of time occurring between blood extraction and blood processing in LeukoLock platforms for each time point (DxGP). The higher this period of time was, the higher the RNA was degraded (lower values of RIN). Therefore, we proposed to include RIN values as a fixed effect or as a covariate in the statistical model used to analyze expression differences. In fact, the same results were obtained using both approaches (data not shown).

RIN values affect Cq of samples depending on amplicon size [44]. The higher the amplified DNA fragment is, the higher the probability is to be broken down. The length of the amplified product was more correlated with RIN values than expected. Amplicon sizes were in the range of 70 to 250 bp (Table 3) for which Fleige and Pfaffl [26] indicates a more or less independence of qPCR products and RNA quality. Furthermore, DNA CG content did not seem to affect RNA stability as it has been previously described, where lower CG degree content was correlated with higher RIN values [45]. *SDHA* amplicon was the one with the highest CG content (65%) but was the most affected by RNA degradation. Only for *MDH1,* which has the lowest CG content and a high RIN effect, this relationship seems to be true. These results reveal that the effect of RNA integrity over both the GOI and the HK should be taken into account in expression analyses.

### HK Selection

A crucial aspect revealed in this work, is the need to test the stability of the candidate HKs and the GOI simultaneously. *MDH1* and *SDHA* were previously selected [38] among 16 candidates tested, as the most stable pair in similar conditions to those evaluated here. *HSP90AA1* was not included in that experiment. In the present work, we have verified that the GOI, *HSP90AA1*, is much more stable than the two previously selected HKs, *MDH1* and *SDHA*. Therefore, none of them can be used to normalize the GOI expression data. The constitutive counterpart of the *HSP90AA1* gene, *HSP90AB1*, showed the best stability values within and between treatments (Table 4), and it was chosen as the HK to normalize the expression data of the *HSP90AA1*. Differences in the stability of both chaperone genes might be due to the effect of the SNPs existing at the promoter of the *HSP90AA1* [13] [40] and to the more inducible behavior of this gene.

### qPCR Experimental Design and Statistical Methods for Expression Data Analyses

When a great number of samples and treatments are included in a qPCR study the experimental design is important since qPCR plates have a limited capacity (96 or 384 wells). In our design, plates contained a randomized set of animals, treatments, genotypes, RIN values and genes to avoid estimation biases. The repetition of one or more samples in all plates connects the plate’s system allowing to remove technical nuisance from this source of variability and to compare results from all plates. We have confirmed that the plate effect is an important source of variability since differences in Cq among plates can reach values up to 1.4.

Traditional statistical methods to analyze qPCR data was restricted to pair-wise comparisons of treatments in which expression data from GOIs are previously normalized with one or more HKs. This kind of approach does not include systematic nor random effects and their interactions that could affect expression results. In the linear mixed model used in this study, GOI and HKs data are simultaneously analyzed.This model includes different sources of biological and technical variation (i.e. plate, RIN, genotypes, genes, and interactions among them) as fixed or random effects. Fitting this model let us checking HK stability, normalization of GOI data with the most stable HK(s) and test the linear hypothesis of the existence of different expression levels of the *HSP90AA1* gene depending on the genotype of the mutations located at its promoter and on diverse environmental conditions.

### Environmental Conditions and Gene Expression

Sheep are believed to be one of the most resistant species to climatic extremes, especially to high environmental temperatures. Environmental conditions in Ciudad Real often exceed sheep thermo neutral zone which is comprised between 5°C and 25°C [46]. As expected, expression results differed between heat stress and control conditions. However, unexpectedly, differences in expression rate among genotypes were observed in samples collected in August but not in July. The scarce differences in climatic parameters existing between August and July collects did not explain the observed differences between these time points in terms of FC. The higher THImax values at collection time and during 5 days before collection in August than in July could be the clue to such differences. Other environmental factors here unknown such as wind speed, number of hours over the comfort temperature, insulation, etc. included in Fanger’s comfort equation [47] would have also contributed in such differences.

Significant differences among A/C_−601_-A/G_−522_-A/G_−444_ and G/C_−660_ among genotypes, found for August and control samples but not for July ones would be explained by the existence of a transition in the expression state of the gene between the basal transcription and the heat stress response. Changes in the expression ranking of G/C_−660_ observed between control and August samples, and also for other SNPs in a less evident way, would support this hypothesis. Also, since the heat stress response is not a permanent state, in terms of gene expression, even when heat shock conditions are still present, acclimatization processes cannot be discarded as possible source of differences found in samples collected in July and August [48].

### Expression Analysis and Genotype Comparisons

Our initial hypothesis was that differences in the expression rate of the gene with different G/C_−660_ genotypes would be observed only under heat stress conditions [24]. Surprisingly, after including a higher amount of samples and a set of 6 additional polymorphisms located also at the *HSP90AA1* promoter, the existence of expression differences under non heat stress situations was confirmed as well. Thus, polymorphisms located in the promoter of *HSP90AA1* affect not only its expression rate as response to heat shock but also its basal transcription levels.

Differences in the expression rate found for the contrasts among alternative genotypes for the SNPs studied here suggest that the transcription of this gene may be multiply regulated by cross-talk of various transcription factors, as it was pointed out for this gene in human [39]. Although much of the heat-induced gene expression can be explained by HSF1, a perfect correlation between its binding and induction has not been found [12]. Signal transduction cascades activated by p53, Jak and Ras pathways via HSF1 binding to the heat-shock response element (HSE) and integrating to modulate HSP transcription have been reported [49]. Additional positive or negative factors may modulate the transcriptional induction of HSF1-bound genes. Moreover, eukaryotic gene expression is tightly regulated at many levels, and can vary its regulation complexity [50]. The core promoter (TATA box, initiator –INR- and downstream promoter element –DPE-), is the essential part. Next to the core, proximal enhancers as cis-control elements (i.e. CCAAT box, GC box, B recognition element (BRE) and STRE elements) might be acting. Upstream, distal enhancers (hormone responsive elements -HRE- and nuclear factor element –NFE-) and a huge diversity of regulators that recruit a cascade of more transcription factors contribute to gene transcription regulation [51], [52]. The SNPs studied in this work are located enough upstream to the beginning of the transcription initiation to consider them as binding sites of these co-regulators, or distal enhancers that do not directly activate the transcription of the gene but modulate its expression.

The role of the SNP G/C_−660_ in the transcription of the gene under heat stress has been confirmed through analysis in which only this mutation is tested. However, results from the analyses of composed genotypes G/C_−660_-A/C_−601_-A/G_−522_-A/G_−444_ and A/C_−601_-A/G_−522_-A/G_−444_ revealed a cooperative relationship among several SNPs in terms of transcription efficiency. Thus, alternative genotypes of SNP_−660_-SNP_−444_ seem to affect the expression of the gene in response to heat stress and those of SNP_−660_-SNP_−522_ the basal transcription of *HSP90AA1*, which may occur under climatic conditions comprising comfort temperatures. Under heat stress conditions, the superiority of CC_−660_ over GC_−660_ and GG_−660_ and of GC_−660_ over GG_−660_ indicated an additive effect for this mutation. However, for the control samples, the GC_−660_ genotype was superior to CC_−660_ and GG_−660_. The effect of the SNP_−444_ was less clear due to the low frequencies of the AG_−444_ and AA_−444_ genotypes; however, for the SNP located at −522, more clear conclusions can be extracted.

Several putative TFs have been predicted (Table S7 and Table S8) for the presence of C and A at SNPs −660 and −444, respectively. Some TFs that could co-activate gene expression as distal enhancers only with CC_−660_ were NFI/CTF (Nuclear factor I or CCAAT box-binding transcription factor) and VDR (Vitamin D receptor) together with RxR-alpha (Retinoid X receptor Alpha). The last two TFs form a heterodimer which attracts a complex of co-activators proteins. This complex links the heterodimer to the initiation complex formed at the TATA box, promoting the transcription machinery [53]. Both TFs bind putatively at the sequence around the SNP_−660_, and VDR only when this position is C. For AG_−444_ a heat shock element that could bind a heat shock factor was predicted for the presence of the A nucleotide [39].

The presence of an INDEL of two adenines (AA) located at position -704 in the promoter [21] completely linked, at least in this breed with SNP_−660_, must also be considered in the expression regulation of the gene under heat stress conditions. Thus, CC_−660_ animals are also homozygous for the AA insertion (AA/AA), animals with GC_−660_ are heterozygous ins/del (AA/−) and animals with GG_−660_ are homozygous for the AA deletion (−/−). This INDEL (AA) was located within a putative glucocorticoid receptor (GR) transacting factor binding site. The AA deletion (−/−) created a GR transcription site. It has been pointed out that glucocorticoids can suppress the heat shock response in stressed cells by inhibiting the action of the heat shock factor 1 (HSF1) [54]. Therefore, this mutation would be the responsible of the expression differences observed for SNP_−660_. Because of the high linkage disequilibrium between the SNPs at −660 and −528 (R^2^ = 0.95) the possible effect of the SNP_−528_ over the transcription rate of the gene under heat stress conditions is masked by the first, and therefore no conclusions could be extracted from this position.

Under control conditions, A/G_−522_ seems to have a predominant effect over the transcription rate of the gene, being AG_−522_ (AA_−522_ was not found in these samples) superior than GG_−522_. Due to the proximity of this mutation to the SNPs located at −528 (5pb) and −524 (1pb) TF binding sites were predicted for a sequence containing the three SNPs (Table S7 and Table S8). Several putative TF binding sites linked to the presence of adenine at −522 and thymine at −524 were found. Among them, the stress response element (STRE) [39] and the JunD (functional component of the AP1 -activator protein 1- transcription factor complex) related with transcription coactivator activity, oxidative stress response [55] and spermatogenesis [56] seemed to be closer to the *HSP90AA1* functions. The TF c-Fos stimulates transcription of genes containing AP-1 regulatory elements and was predicted for the sequence AtagTcA for the SNPs at −528, −524 and –522. In our samples animals with AG_−522_ were always CC-_601_-TT_−524_-TT_−468_ and in most cases (70%) CC-_601_-AG_−528_-TT_−524_-TT_−468_. Two putative TF binding sites implying the presence of cytosine were found for A/C_−601_, HES-1 (hairy and enhancer of split-1) [57], which can act as a repressor or activator, and USF1 (Upstream stimulatory factor 1) [58], that has been found to be involved in the stress-activated signaling cascade [59] and in the cessation of Sertoli cell proliferation and differentiation to spermatozoids [60]. For the SNP_−468_ one interesting homolog of the human ZNF395 binding Sp1 was found for maize [61] linked to the response to oxidative stress (Table S7).

Most stress-response genes are regulated in a concordant manner with respect to transcript levels and translational efficiency. A strong overall correlation has been observed between transcriptional/translational induction of genes and induction of the corresponding proteins [62]. During environmental stress in fission yeast, most mRNAs are regulated both at transcription and translation level but only up-regulated mRNAs showed a strong correlation with protein expression, while down-regulated mRNAs showed no such correlation [62]. Therefore changes in the expression rate of the *HSP90AA1* here observed as a function of environmental temperatures and genotypes at the SNPs located in its promoter will be accompanied by changes in the amount of protein produced. Thus, those genotypes which showed higher expression levels under heat shock also will display higher protein amounts. Despite the low magnitude of the changes in *HSP90AA1* expression rate observed, even a small proportion may be significant as HSP90 is one of the most abundant proteins in most cells [39]. Higher amounts of HSP90α protein would increase its capacity to exert its protective role over the effects caused by heat stress at the cellular level. Effects of changes in the protein amount as consequence of several stress sources must be studied in tissues in which HSP90α predominates, such as brain and testis [39]. In this context, our results for two of the known functions of the HSP90α, refolding proteins with aberrant conformation [39] and spermatogenesis and meiotic progression in testis [63], confirm this hypothesis. GG_−660_ was related with higher sperm DNA fragmentation values in rams under heat stress conditions [23] and also to lower scrapie incubation period in sheep (missfolding stress) [22].

Future studies will focus on testing TF interaction at binding sites where polymorphisms of the *HSP90AA1* promoter are located, by employing in vitro techniques of EMSA (Electrophoretic Mobility Shift Assay). In addition, an epigenetic regulation of the *HSP90AA1* expression cannot be discarded. Methylation of CpG islands located at gene promoters is a well known mechanism of expression regulation and gene silencing [64]. A CpG island has been predicted for the promoter of this gene and some of the polymorphisms here analyzed are susceptible to be methylated (SNPs located at −660, −601, −528 and −522). Therefore, future research will be also focused in the study of the methylation pattern of alternative genotypes of the SNPs containing cytosine by bisulfite sequencing techniques.

## Supporting Information

Figure S1 **Sequence and polymorphisms of the ovine** ***HSP90AA1*** **gene promoter (DQ983231).** Intron sequence in lower case and exons in capital letters. Primers sequences used to amplify the 499pb fragment are highlighted in dark. SNPs are in square brackets. The 7 SNPs of interest included in the 499pb amplicon sequenced (−660, −601, −528, −524, −522 and −444) are in grey. INDELs are in brackets. Putative methylated SNPs are also circled. Initiation of transcription (TATA box) and translation (ATG) in bold. A HSE already detected is underlined. Modified from Marcos-Carcavilla and coworkers [20].
(TIF)

Figure S2 **Fold change (FC) for the contrast among alternative genotypes G/C_−660_-G/A_−522_ of the** ***HSP90AA1*** **promoter within each treatment (Control, July and August) normalized by** ***HSP90AB1.*** Segments indicate the 95% confidence interval (FC_up_-FC_low_). In abscissa the FC, in ordinate genotype contrasts. Asterisk over each bar indicates the significance level of the contrasts.
(TIF)

Figure S3 **Fold change (FC) for the contrast among alternative genotypes A/C_−601_-G/A_−522_ of the** ***HSP90AA1*** **promoter within each treatment (Control, July and August) normalized by** ***HSP90AB1.*** Segments indicate the 95% confidence interval (FC_up_-FC_low_). In abscissa the FC, in ordinate genotype contrasts. Asterisk over each bar indicates the significance level of the contrasts.
(TIF)

Table S1 **SNP frequencies of the polymorphisms found at the** ***HSP90AA1*** **promoter as described in Marcos-Carcavilla’s work** [**24**] **.**
(XLSX)

Table S2 **Genotype contrasts of G/C_−660_-A/C_−601_-A/G_−522_-A/G_−444_.** Estimates, standard error (SE), t values and p values of significant contrasts among genotypes G/C_−660_-A/C_−601_-A/G_−522_-A/G_−444_ of the *HSP90AA1* gene promoter normalized with *HSP90AB1* in each treatment (Control, July and August). Also Fold change (FC) and the 95% FC confidence interval (FC_up_-FC_low_) are included.
(XLSX)

Table S3 **Genotype contrasts of A/C_−601_-A/G_522_-A/G_−444_.** Estimates, standard error (SE), t values and p values of significant contrasts among genotypes A/C_−601_-A/G_522_-A/G_−444_ of the *HSP90AA1* gene promoter normalized with *HSP90AB1* in each treatment (Control, July and August). Also Fold change (FC) and the 95% FC confidence interval (FC_up_-FC_low_) are included.
(XLSX)

Table S4 **Genotype contrasts of SNP G/C_−660_.** Estimates, standard error (SE), t values and p values of significant contrasts among genotypes G/C_−660_ of the *HSP90AA1* gene promoter normalized with *HSP90AB1* in each treatment (Control, July and August). Also Fold change (FC) and the 95% FC confidence interval (FC_up_-FC_low_) are included.
(XLSX)

Table S5 **Hardy-Weinberg equilibrium analysis of the 7SNPs of interest at** ***HSP90AA1*** **gene promoter on trios population.**
(XLSX)

Table S6 **LD analyses from trios population with PLINK software.** Linkage disequilibrium values between each couple of SNPs are shown. The couples of SNPs in bold show a high degree of linkage disequilibrium between them.
(XLSX)

Table S7 **Predicted transcription factor binding sites from TESS software.**
(XLSX)

Table S8 **Predicted transcription factor binding sites from ALGENN-PROMO software.**
(XLSX)
