# Supplementary figures and images for: Ovine HSP90AA1 Expression Rate Is Affected by Several SNPs at the Promoter under Both Basal and Heat Stress Conditions
Source: PLoS One. 2013 Jun 24;8(6):e66641. doi: 10.1371/journal.pone.0066641 (PMC3691178; doi:10.1371/journal.pone.0066641)

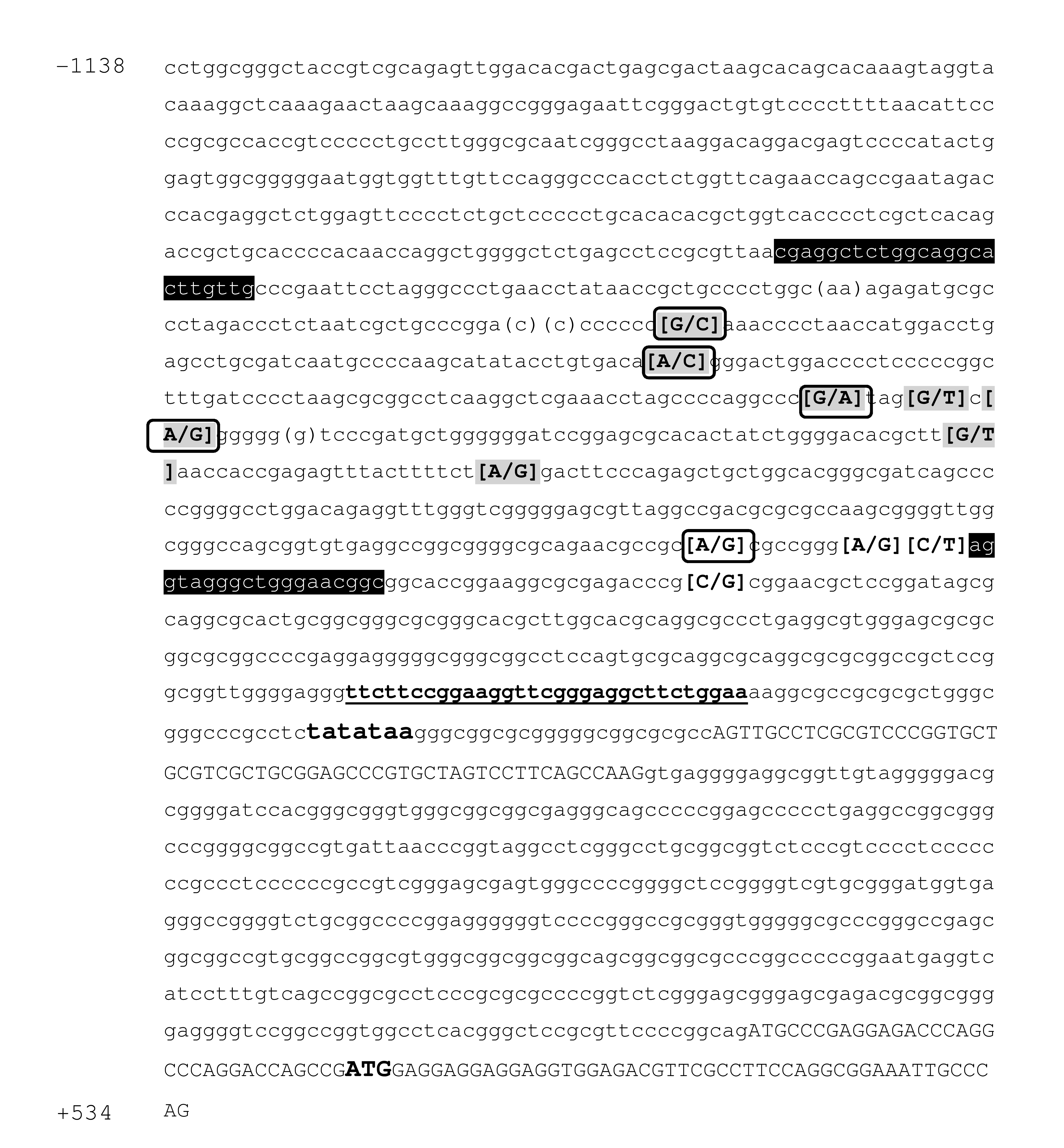

Supplement: Figure S1 — Sequence and polymorphisms of the ovine HSP90AA1 gene promoter (DQ983231). Intron sequence in lower case and exons in capital letters. Primers sequences used to amplify the 499pb fragment are highlighted in dark. SNPs are in square brackets. The 7 SNPs of interest included in the 499pb amplicon sequenced (−660, −601, −528, −524, −522 and −444) are in grey. INDELs are in brackets. Putative methylated SNPs are also circled. Initiation of transcription (TATA box) and translation (ATG) in bold. A HSE already detected is underlined. Modified from Marcos-Carcavilla and coworkers [20]. (TIF) [file pone.0066641.s001.tif]

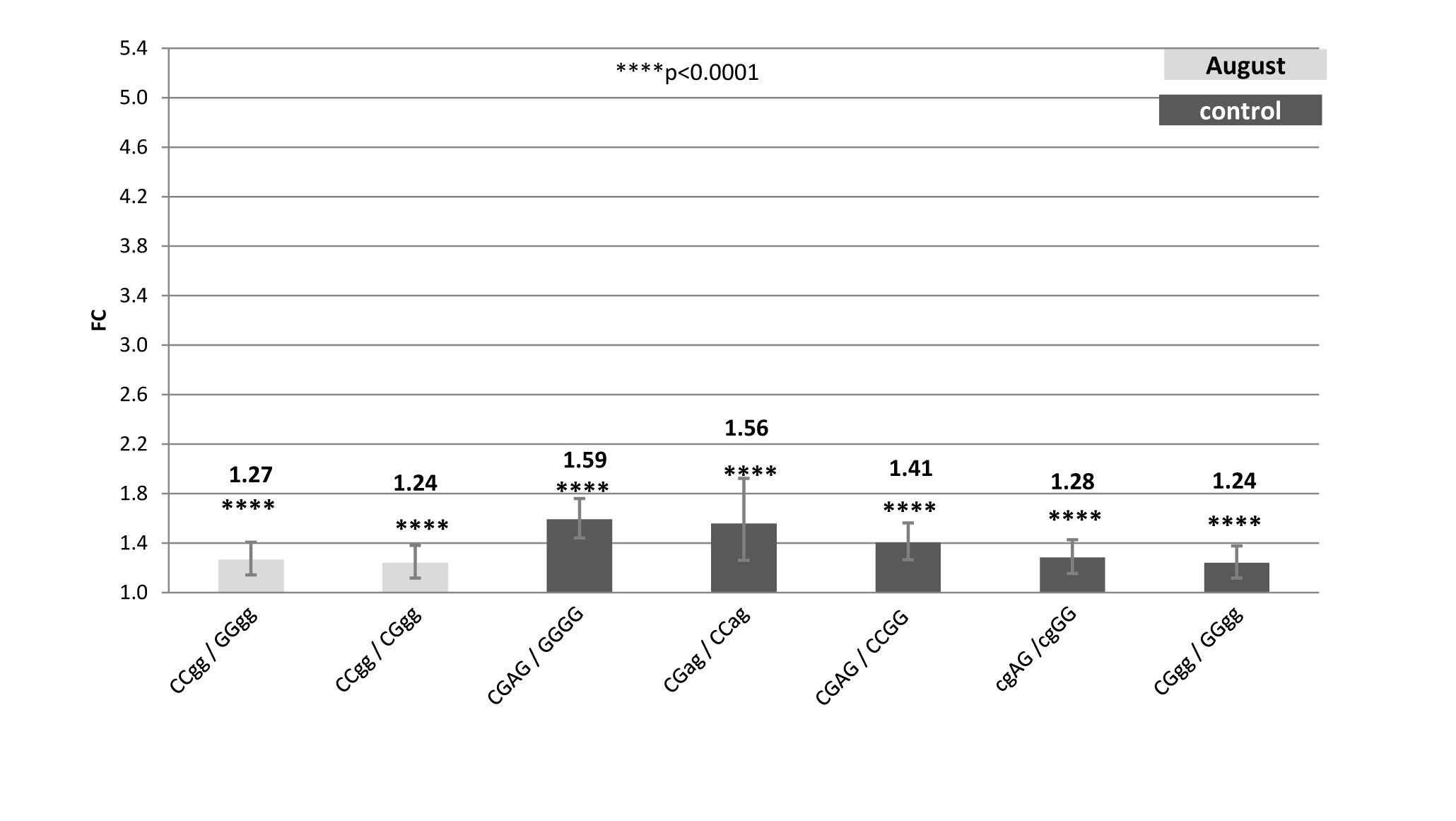

Supplement: Figure S2 — Fold change (FC) for the contrast among alternative genotypes G/C−660-G/A−522 of the HSP90AA1 promoter within each treatment (Control, July and August) normalized by HSP90AB1. Segments indicate the 95% confidence interval (FCup-FClow). In abscissa the FC, in ordinate genotype contrasts. Asterisk over each bar indicates the significance level of the contrasts. (TIF) [file pone.0066641.s002.tif]

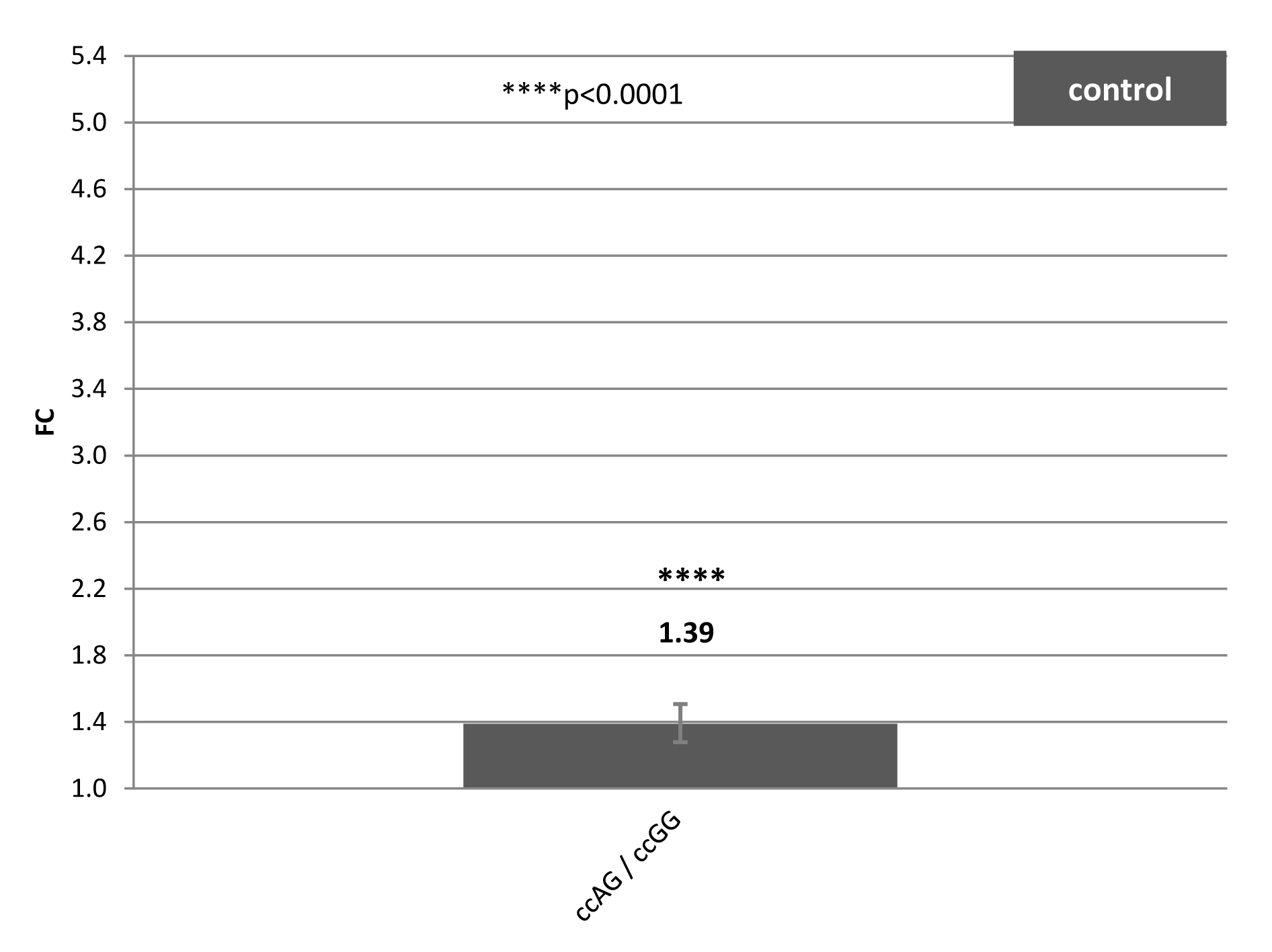

Supplement: Figure S3 — Fold change (FC) for the contrast among alternative genotypes A/C−601-G/A−522 of the HSP90AA1 promoter within each treatment (Control, July and August) normalized by HSP90AB1. Segments indicate the 95% confidence interval (FCup-FClow). In abscissa the FC, in ordinate genotype contrasts. Asterisk over each bar indicates the significance level of the contrasts. (TIF) [file pone.0066641.s003.tif]
